# Effect of informed consent on patients undergoing gastrointestinal surgery and living donor liver transplantation and on their relatives in a developing country

**DOI:** 10.1002/bjs5.37

**Published:** 2018-02-07

**Authors:** S. Ray, N. N. Mehta, S. Mehrotra, S. Lalwani, V. Mangla, A. Yadav, S. Nundy

**Affiliations:** ^1^ Department of Surgical Gastroenterology and Liver Transplantation Sir Ganga Ram Hospital New Delhi India

## Abstract

**Background:**

Informed consent is a systematic process for obtaining permission before conducting a healthcare intervention. In a developing country, gaining informed consent is generally perceived to be a ritual only to comply with legal requirements. The present study examined this by assessing the process of informed consent in patients undergoing gastrointestinal surgery or living donor liver transplantation (LDLT) and their relatives, based on their comprehension and overall satisfaction, in India.

**Methods:**

All patients undergoing any gastrointestinal surgery or LDLT procedure between August 2015 and July 2016 and their relatives were included, and were administered a structured questionnaire 5 days after the procedure.

**Results:**

The majority of patients (94·2 per cent) could recall the nature of their disease, the surgery performed (81·6 per cent) and anticipated complications (55·6 per cent). Among their relatives, these proportions were 97·8, 87·3 and 58·5 per cent respectively. Recall was associated with age, occupation and education among both patients and relatives. Patients undergoing LDLT, their donors and their relatives had better recall than those who had other gastrointestinal procedures (P < 0·001). Many patients found the process of informed consent useful and reassuring.

**Conclusion:**

The details and risks of an operation were understood by most of the patients, especially those undergoing liver transplantation. Patients from developing countries can generally understand ‘informed consent’, and value it.

## Introduction

Informed consent is the systematic process for obtaining permission from a person before conducting a healthcare intervention on them^1^. In many countries it is a legal obligation for the treating surgeon to explain fully to the patient the nature of the proposed operation, the risks involved, and the alternative treatment available in a language that he or she clearly understands.

In a developing country such as India, where the majority of the population is poor and illiterate, the process of obtaining informed consent has been perceived to be mainly a meaningless legal ritual during which a patient is presented with information that is usually beyond his or her understanding^2^. The present study investigated whether this perception of the patients' inability to comprehend the informed consent process was actually correct. Both patients and their relatives were included in this study.

## Methods

### Patients and setting

The study was conducted in the Department of Surgical Gastroenterology and Liver Transplantation, Sir Ganga Ram Hospital, New Delhi, India, between August 2015 and July 2016. The hospital is one of the largest tertiary care centres in India. The process of informed consent is legally obligatory in India. Owing to patriarchal societal norms and strong family bonds, patients usually prefer the presence of a close family member at the time of decision‐making. All patients who were 18 years or older and who underwent gastrointestinal surgery or liver transplantation were eligible for inclusion. Younger patients, patients undergoing day care procedures, patients who were mentally not capable of answering questions, and patients in whom the power of attorney had been vested upon some other family member were excluded from the study.

At the outpatient clinic, a consultant explained the nature of their disease, the procedure contemplated, alternative options available and the risks associated with the operation. Thereafter, detailed consent was explained to the patient as well as one of the decision‐making relatives by the senior resident on duty the evening before the surgery in case of elective operations and just before emergency procedures. A special liver transplant form (according to institutional protocol) was used in addition to the routine informed consent form for donors and recipients undergoing living donor liver transplantation. Information included the nature of the disease, organ system affected, nature of surgery, major and minor complications, possible alternative treatment options available, need for an additional intraoperative intervention including the possible creation of a stoma, and the need to photograph the operation for the hospital record and for academic interest. The consent was explained in English and Hindi. For foreign nationals, interpreters were used whenever required. The patient and their relative were always seen together by the surgeon explaining the informed consent. As this was the norm followed in the hospital across most of the departments and did not involve any form of added investigation or intervention, approval of the institutional review board was not considered necessary.

### Questionnaires

Two sets of detailed questionnaires were handed over to the patient and the relative on the fifth day after surgery (*Appendix S1*, supporting information). The first comprised ten questions with a single best response to assess recall, and the second comprised a set of ten points in a feedback assessment format to be answered on a scale of 0–10, with 0 being the worst and 10 the best. This was to assess the satisfaction of the patient or relative with the process of informed consent. The questionnaires were designed by the investigators based on the objective points addressed by the hospital's informed consent sheet. Illiterate patients and relatives were asked to answer the questions verbally in the presence of the resident collecting the questionnaire sheet. As the questions were objective and involved single best responses in most instances, bias was avoided as much as possible.

The recall elements were graded as none (score less than 4), partial (score 4–7) or complete (score 8–10). This denotes overall recall, which was the sum of all the individual elements of the recall questionnaire. Similarly, overall satisfaction was graded as dissatisfied (score of 4 or less), partly satisfied (score 5–7) and highly satisfied (score 8–10). The data were tabulated by the principal investigator.

### Statistical analysis

The aim was to include all possible patients and relatives over the study period. Approximately 500 patients and corresponding relatives were expected to participate in the study. All statistical analyses were performed with SPSS^®^ version 20.0 (IBM, Armonk, New York, USA). Categorical variables were presented as number and percentage, and analysed with the χ^2^ test. Comparison of the patients and relatives and liver transplant patients *versus* the other gastrointestinal surgical patients was done using the Mann–Whitney *U* test and the Wilcoxon signed rank test. Association between age, sex, occupation, educational status, and recall ability and satisfaction was determined with the Pearson χ^2^ test. Incomplete questionnaires were excluded from analyses. Recall of liver transplant patients and relatives was compared with that of the other patients and their corresponding relatives. *P* < 0·050 (two‐sided) was considered statistically significant.

## Results

### Demographics

**Figure 1:**
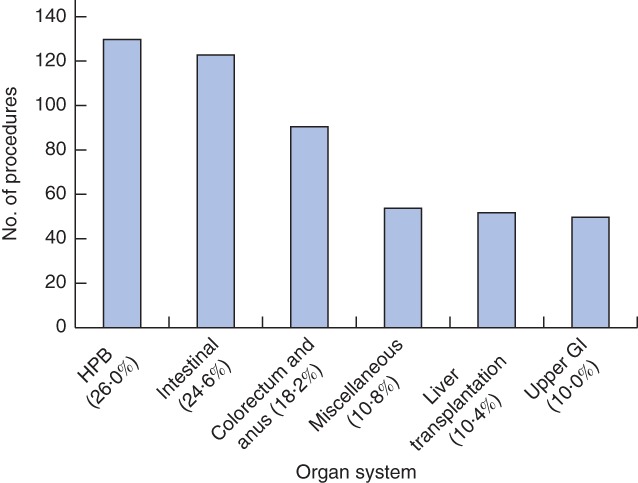
Total number of surgical procedures performed per subspecialty during the study period. HPB, hepatopancreatobiliary; GI, gastrointestinal

One thousand individuals (500 patients and 500 relatives) filled out the questionnaires. Their demographics are shown in *Table* 1. The median age of the population was 45 (range 3–90) years. Surgical procedures are shown in *Fig*. 1. Some 86 patients, who were too unwell to participate in the postoperative period, and four relatives, who were not available, were excluded from the final analyses.

**Table 1:** Demographics of patients and their relatives

	No. of patients (*n* = 500)	No. of relatives (*n* = 496)
Age (years)		
≤ 50	355 (71·0)	401 (80·8)
> 50	145 (29·0)	95 (19·2)
Sex		
M	303 (60·6)	294 (59·3)
F	197 (39·4)	202 (40·7)
Education		
Illiterate	15 (3·0)	6 (1·2)
Primary school	65 (13·0)	10 (2·0)
High school	127 (25·4)	110 (22·2)
Graduate	293 (58·6)	370 (74·6)
Occupation		
Student	37 (7·4)	27 (5·4)
Service	118 (23·6)	211 (42·5)
Business	126 (25·2)	122 (24·6)
Miscellaneous	219 (43·8)	136 (27·4)
Excluded/drop‐outs	86 (17·2)*	4 (0·8)†

Values in parentheses are percentages.

* Includes patients who were unwell in the postoperative period or on ventilator support.

† Includes patients who did not have a relative to answer the respective questionnaire.

### Recall of informed consent

Analysis of recall of the various components of informed consent, categorized as recall of the diagnosis, surgery and complications, and the overall recall score, is shown in *Table* 2. Most respondents could completely recall the primary diagnosis, the nature of the surgery and associated complications. The overall recall score was 8 or more in most patients (248 of 414, 59·9 per cent) and relatives (306 of 496, 61·7 per cent). The recall score correlated significantly with age, educational status and occupation (*Table* 3).

**Table 2:** Recall among 414 patients and 496 relatives

	No recall	Partial recall	Complete recall
Recall diagnosis			
Patients	7 (1·7)	17 (4·1)	390 (94·2)
Relatives	2 (0·4)	9 (1·8)	485 (97·8)
Recall surgery			
Patients	16 (3·9)	60 (14·5)	338 (81·6)
Relatives	2 (0·4)	61 (12·3)	433 (87·3)
Recall complications			
Patients	34 (8·2)	150 (36·2)	230 (55·6)
Relatives	13 (2·6)	193 (38·9)	290 (58·5)
Overall recall			
Patients	14 (3·4)	152 (36·7)	248 (59·9)
Relatives	4 (0·8)	186 (37·5)	306 (61·7)

Values in parentheses are percentages.

**Table 3:** Association of recall with demographic variables in 414 patients and 496 relatives

	No recall	Partial recall	Complete recall	*P* *
Age (years)				
Patients				0·022
≤ 50	5 (1·9)	55 (20·6)	207 (77·5)	
> 50	12 (8·2)	67 (45·6)	68 (46·3)	
Relatives				0·031
≤ 50	4 (1·2)	68 (21·1)	250 (77·6)	
> 50	9 (5·2)	65 (37·4)	100 (57·5)	
Sex				
Patients				0·152
M	2 (0·8)	74 (30·7)	165 (68·5)	
F	3 (1·7)	67 (38·7)	103 (59·5)	
Relatives				0·152
M	3 (1·0)	105 (35·7)	186 (63·3)	
F	3 (1·5)	80 (39·6)	119 (58·9)	
Education				
Patients				0·001
Illiterate	5 (45)	3 (27)	3 (27)	
School	11 (7·6)	48 (33·3)	85 (59·0)	
Graduate	0 (0)	18 (6·9)	241 (93·1)	
Relatives				0·002
Illiterate	1 (50)	1 (50)	0 (0)	
School	3 (2·5)	71 (58·7)	47 (38·8)	
Graduate	1 (0·3)	114 (30·6)	258 (69·2)	
Occupation				
Patients				0·001
Service	0 (0)	21 (20·4)	82 (79·6)	
Business	1 (1)	24 (35)	44 (64)	
Other	11 (4·5)	73 (30·2)	158 (65·3)	
Relatives				0·022
Service	1 (0·5)	62 (29·4)	148 (70·1)	
Business	1 (0·9)	52 (46·4)	59 (52·7)	
Other	3 (1·7)	72 (41·6)	98 (56·6)	

Values in parentheses are percentages.

* χ^2^ test.

### Satisfaction with the informed consent process

The satisfaction of patients and relatives with the informed consent process in terms of usefulness, relevance and clarification of doubts is shown in *Table* 4. More than 80 per cent of the patients and relatives found the informed consent process very useful. More than 75 per cent found it relevant to the clinical scenario and effective in clearing their doubts and misconceptions regarding the disease and the intervention that had been proposed and executed. Approximately 60 per cent of the patients and relatives were satisfied with the overall process of informed consent.

**Table 4:** Satisfaction with the informed consent process among 414 patients and 496 relatives

	Dissatisfied	Partly satisfied	Fully satisfied
Usefulness			
Patients	12 (2·9)	60 (14·5)	342 (82·6)
Relatives	7 (1·4)	70 (14·1)	419 (84·5)
Relevance			
Patients	15 (3·6)	76 (18·4)	323 (78·0)
Relatives	12 (2·4)	100 (20·2)	384 (77·4)
Clarification of myths/doubts			
Patients	10 (2·4)	78 (18·8)	326 (78·7)
Relatives	10 (2·0)	120 (24·2)	366 (73·8)
Overall satisfaction			
Patients	4 (1·0)	158 (38·2)	252 (60·9)
Relatives	3 (0·6)	206 (41·5)	287 (57·9)

Values in parentheses are percentages.

The level of satisfaction correlated with educational status, with graduates being more satisfied than the rest in both groups (*Table* 5). More than 60 per cent of those in service occupations were highly satisfied with the informed consent, and this was found to be statistically significant.

**Table 5:** Association of satisfaction with demographic variables in 414 patients and 496 relatives

	Dissatisfied	Partly satisfied	Highly satisfied	*P* *
Age (years)				
Patients				0·135
≤ 50	5 (1·9)	100 (37·5)	162 (60·7)	
> 50	6 (4·1)	54 (36·7)	87 (59·2)	
Relatives				0·135
≤ 50	2 (0·6)	80 (24·8)	240 (74·5)	
> 50	6 (3·4)	45 (25·9)	123 (70·7)	
Sex				
Patients				0·124
M	4 (1·7)	61 (25·3)	176 (73·0)	
F	3 (1·7)	51 (29·5)	119 (68·8)	
Relatives				0·120
M	1 (0·3)	120 (40·8)	173 (58·8)	
F	2 (1·0)	86 (42·6)	114 (56·4)	
Education				
Patients				0·025
Illiterate	1 (9)	4 (36)	6 (55)	
School	11 (7·6)	66 (45·8)	67 (46·5)	
Graduate	0 (0)	88 (40·0)	171 (60·0)	
Relatives				0·001
Illiterate	0 (0)	0 (0)	2 (100)	
School	2 (1·7)	30 (24·8)	89 (73·6)	
Graduate	0 (0)	31 (8·3)	342 (91·7)	
Occupation				
Patients				0·013
Service	0 (0)	38 (36·9)	65 (63·1)	
Business	0 (0)	22 (32)	47 (68)	
Other	10 (4·1)	98 (40·5)	134 (55·4)	
Relatives				0·042
Service	0 (0)	76 (36·0)	135 (64·0)	
Business	1 (0·9)	54 (48·2)	57 (50·9)	
Other	2 (1·2)	76 (43·9)	95 (54·9)	

Values in parentheses are percentages.

* χ^2^ test.

No difference was observed in overall recall ability and level of satisfaction between patients and relatives (*Table S1*, supporting information).

Patients undergoing liver transplant operations and their relatives had better recall ability and were more satisfied with the informed consent process than the patients undergoing gastrointestinal surgery and their relatives (*Table S2*, supporting information).

## Discussion

In this study, patients and relatives in a developing country understood informed consent well and valued it. Despite their varying levels of education and socioeconomic status, the general perception was that the informed consent process was comprehensible and useful^3^.

For the consent to be valid, the patient must be competent enough to take a decision, should not be under undue stress, and must be presented with true and sufficient information about the intervention^4^. This appeared to be the case in this study, and demonstrates that in a developing country such as India the informed consent process is there not just for the doctor but also for the patient. These results are in contrast to expectations. In a paternalistic society such as India, concerns regarding subordination of the patients' interests to the competing interests of the family also exist. Typically, the decision is guided by the oldest male member of the family, side‐lining the decision of the patient undergoing the intervention, who may be wise and competent enough to make his or her own choice^4^.

Most complaints in the Western world are provoked by doctors failing to communicate adequately with patients regarding the surgical procedure^5^. Another point that often arises in the context of informed consent is the extent of detail that needs to be conveyed to the patient^6^. With the improvement in India's literacy rates, from 12 per cent in the preindependence era to 74 per cent in 2011 (2013 census), it becomes important to assess any significant change in comprehension of informed consent from the past results^7^.

Recall of the primary diagnosis and surgical procedure was better than that of the major and minor complications associated with the treatment. From a study of 100 consecutive patients undergoing elective abdominal operations, Sanwal and colleagues^8^ reported an overall recall rate by patients of 70 per cent, compared with complete recall of diagnosis and surgical procedures of about 85 per cent among patients and relatives in the present study. In fact, the results of the present study were even better than those found in some studies reported from Western countries. Fortun and co‐workers^9^ evaluated 82 healthy volunteers undergoing capsule endoscopy regarding their recall of the information provided about the procedure, and also compared the difference between the medically trained and the non‐medical participants. Only 17 per cent could recall three or more major risks associated with the procedure, and only 12 per cent could identify the names of the three trial drugs used. Most participants (90 per cent) could recall the main procedure and the most common associated risk (64 per cent). Another study^10^ showed that only 50 per cent of the patients were aware of the basic facts relating to the abdominal operation on postoperative days 2–5.

Nearly 90 per cent of patients and their relatives in the present study comprehended the information and were partly or fully satisfied with the process of informed consent. The findings are concordant with reports from Western countries. Pope *et al*.^11^ reported a 95–98 per cent rate of satisfaction by the respondents with the process of informed consent.

According to the recommendation of the Association of American Medical Colleges, three strategies to improve the process of informed consent are ensuring short and simple information, dividing the information into essential and supplemental sections, and verifying patient comprehension^12^. Paasche‐Orlow and colleagues^13^ showed that Institutional Review Boards commonly provide text for informed consent forms that fall short of their own readability standards. The present study used a simple and comprehensively structured two‐page consent form, explaining the information in English and Hindi to ensure better understanding of the process.

This study did not observe a significant difference in recall ability and satisfaction among the patients and their relatives. The treating clinician informed both a close relative and the patient during the same process. This may enhance effective and adequate communication to patient and family. Liver transplant patients and their relatives had better recall of the informed consent process and were more satisfied than the other patients and their corresponding relatives. This may be caused by different demographics, more detailed information, or timing of the informed consent. Fagerlin and co‐workers^14^ suggested that more detailed communication of the risks to the patient ensured better comprehension. Unlike the other patients, to whom the details of the surgery and associated risks were explained the evening before the operation, the liver transplant donors and recipients had informed consent explained in detail at the time of initial evaluation in addition to the evening before the surgery. Anderson and Wearne^15^ recommend that the ideal time for obtaining informed consent is at the time of listing in the surgical clinic. They found a greater degree of stress and apprehension among patients when the procedure was explained the night before surgery. In addition, the higher cost and more serious nature of the procedure could be other factors contributing to a greater degree of attention to detail by transplant patients and their families.

This study has several limitations: explanation of the informed consent details will have varied between doctors despite using a standard form; the timing of informed consent varied; and there was no control group of patients who did not receive informed consent, as this was considered unethical and illegal.

## Supporting information


**Appendix S1** Final questionnaire for the informed consent project (Sir Ganga Ram Hospital, Surgical Gastroenterology and Liver Transplantation unit I)
**Table S1** Recall and satisfaction among patients and relatives
**Table S2** Recall and satisfaction among the liver transplant group (donors, recipients and relatives) and the other GI surgical patients
