# Supplementary material for: Effect of informed consent on patients undergoing gastrointestinal surgery and living donor liver transplantation and on their relatives in a developing country
Source: BJS Open. 2018 Feb 7;2(1):34–9. doi: 10.1002/bjs5.37 (PMC5989942; doi:10.1002/bjs5.37)
Supplement: Supplementary file 1 — Appendix S1 Final questionnaire for the informed consent project (Sir Ganga Ram Hospital, Surgical Gastroenterology and Liver Transplantation unit I) Table S1 Recall and satisfaction among patients and relatives Table S2 Recall and satisfaction among the liver transplant group (donors, recipients and relatives) and the other GI surgical patients [file BJS5-2-34-s001.docx]

**BJS5_37**

**Effect of informed consent on patients undergoing gastrointestinal surgery and living donor liver transplantation and on their relatives in a developing country**

**S. Ray, N. N. Mehta, S. Mehrotra, S. Lalwani, V. Mangla, A. Yadav and S. Nundy**

**Appendix S1** Final questionnaire for the informed consent project (Sir Ganga Ram Hospital, Surgical Gastroenterology and Liver Transplantation unit I)

**QUESTIONNAIRE 1**

1. What was the diagnosis explained to you preoperatively?

Ans:

1. What organ system of the body was affected?

- Esophagus.
- Stomach.
- Small intestine.
- Large intestine.
- Rectum and anus.
- Liver.
- Gall bladder/bile duct.
- Pancreas.
- Others.

1. Were you explained about any other treatment options available for the same by the doctor? (yes/no)

- Yes.
- No.
- Don’t remember.

1. What surgery was proposed for your treatment?

Ans:

1. Which of the following risk(s) of the proposed surgery was explained to you?

- Bleeding/post op hemorrhage/shock
- Anastomotic leak: enteric/biliary/esophageal.
- Delayed gastric emptying/post op ileus.
- Risk of mortality.

1. Were there any other minor risk(s) explained? (yes/no)

- Yes.
- No.
- Don’t remember.

1. Were you explained about the possibility of ICU admission in post operative course? (yes/no).

- Yes.
- No.
- Don’t remember.

1. Was the need for any additional procedure explained to you in the event of any unforeseen circumstance intraoperatively? (yes/no)

- Yes.
- No.
- Don’t remember.

1. Were you explained about the possibility of a fecal diversion preoperatively? (yes /no).

- Yes.
- Was not applicable to my surgery.
- Was applicable but I was not explained.
- Don’t remember.

1. Were you explained about the need for photographing/videographing the intraoperative details in view of academic purpose, keeping in mind the privacy of your identity?

- Yes and I gave my consent for the same.
- No.
- Don’t remember.

1. Were you explained about the overall cost of your treatment preoperatively? Did it match well with the real cost postoperatively?

Ans:

**QUESTIONNAIRE 2**

**Q1. Give score from 0 to 10 on the utility of understanding of various procedures etc. explained during the informed consent session:**

1. Understanding the diagnosis.
2. Procedure to be done.
3. Possible risks associated with the procedure(s)
4. Alternatives to the procedure
5. Recovery period
6. Possible outcome in case of no treatment

**Q2. Structure of informed consent (tick mark (**√**) the appropriate one)**

1. Highly relevant
2. Relevant
3. Partially relevant and needs improvement
4. Useless and needs thorough modification
5. No need at all
6. Can’t say

**Q3. Clarification of doubts/myths/misconception about the procedure and treatments**

1. Highly useful and helped in restoring confidence
2. Somewhat useful
3. Added unnecessary confusion and nervousness
4. Can’t say

**Q4. Your overall satisfaction level in reference to informed consent session** (Give score from 0 to 10)

**Table S1** Recall and satisfaction among patients and relatives

|  |  | **N** | **Mean Rank** | **P value** |
| --- | --- | --- | --- | --- |
| **Total recall** | Patients | 414 | 448.62 | 0.6 |
|  | Relatives | 496 | 457.55 |  |
| **Usefulness** | Patients | 414 | 463.54 | 0.35 |
|  | Relatives | 496 | 447.89 |  |
| **Relevance** | Patients | 414 | 450 | 0.64 |
|  | Relatives | 496 | 457.32 |  |
| **Clarification** | Patients | 414 | 461.29 | 0.34 |
|  | Relatives | 496 | 447.96 |  |
| **Overall satisfaction** | Patients | 414 | 463.36 | 0.31 |
|  | Relatives | 496 | 446.25 |  |

(Mann-Whitney U test and Wilcoxon W test)

**Table S2** Recall and satisfaction among the liver transplant group (donors, recipients and relatives) and the other GI surgical patients

|  |  | **N** | **Mean Rank** | **P value** |
| --- | --- | --- | --- | --- |
| **Total recall** | Liver transplant | 100 | 608.73 | <0.001 |
|  | Others | 810 | 435.1 |  |
| **Usefulness** | Liver transplant | 100 | 682.69 | <0.001 |
|  | Others | 810 | 428.11 |  |
| **Relevance** | Liver transplant | 100 | 587.74 | <0.001 |
|  | Others | 810 | 438.17 |  |
| **Clarification** | Liver transplant | 100 | 449.51 | 0.825 |
|  | Others | 810 | 454.53 |  |
| **Overall satisfaction** | Liver transplant | 100 | 701.77 | <0.001 |
|  | Others | 810 | 424.67 |  |

(Mann-Whitney U test and Wilcoxon W test)
